# Determination of mono- and diacylglycerols from E 471 food emulsifiers in aerosol whipping cream by high-performance thin-layer chromatography–fluorescence detection

**DOI:** 10.1007/s00216-020-02876-2

**Published:** 2020-08-30

**Authors:** Claudia Oellig, Max Blankart, Jörg Hinrichs, Wolfgang Schwack, Michael Granvogl

**Affiliations:** 1 Department of Food Chemistry and Analytical Chemistry (170a), Institute of Food Chemistry, University of Hohenheim, Garbenstrasse 28, 70599 Stuttgart, Germany; 2 Department of Soft Matter Science and Dairy Technology (150e), Institute of Food Science and Biotechnology, University of Hohenheim, Garbenstrasse 21, 70599 Stuttgart, Germany

**Keywords:** Food emulsifiers, Mono- and diacylglycerols (MAG and DAG), E 471, Aerosol whipping cream, LLE, High-performance thin-layer chromatography–fluorescence detection (HPTLC–FLD)

## Abstract

Mono- and diacylglycerol (MAG and DAG) emulsifiers (E 471) are widely applied to regulate techno-functional properties in different food categories, for example, in dairy products. A method for the determination of MAG and DAG in aerosol whipping cream by high-performance thin-layer chromatography with fluorescence detection (HPTLC–FLD) after derivatization with primuline was developed. For sample preparation, aerosol whipping cream was mixed with ethanol, followed by the addition of water and liquid-liquid extraction with *tert*-butyl methyl ether. The sample extracts were analyzed by HPTLC–FLD on silica gel LiChrospher plates with *n*-pentane/*n*-hexane/diethyl ether (22.5:22.5:55, *v/v/v*) as mobile phase, when interfering matrix like cholesterol and triacylglycerols were successfully separated from the E 471 food additives. For quantitation, an emulsifier with known composition was used as calibration standard and the fluorescent MAG and DAG were scanned at 366/> 400 nm. Limits of detection and quantitation of 4 and 11 mg/100 g aerosol whipping cream were obtained for both monostearin and 1,2-distearin, respectively, and allowed the reliable quantitation of MAG and DAG from E 471 far below commonly applied emulsifier amounts. Recoveries from model aerosol whipping cream with 400 mg E 471/100 g were determined in a calibration range of 200–600 mg E 471/100 g sample and ranged between 86 and 105% with relative standard deviations below 7%. In aerosol whipping creams from the German market, E 471 amounts ranged between 384 and 610 mg/100 g.

**Electronic supplementary material:**

The online version of this article (10.1007/s00216-020-02876-2) contains supplementary material, which is available to authorized users.

## Introduction

Mono- and diacylglycerols (MAG and DAG) are known for their surface-active properties, and thus, frequently used as food emulsifiers (food additive E 471). Their application takes place to adjust techno-functional characteristics such as viscosity, creaming, and foaming stability mainly during the production of bread, pastry, margarines, ice cream, and other dairy products [1]. The composition of the emulsifier directly affects the techno-functional properties of the product and deviations in the relative composition and its dosage distinctly influence product structures, especially viscosity properties [1]. Therefore, a constant composition of the applied emulsifier is essential to guarantee shelf life and high product quality. Deviating product properties of aerosol whipping cream due to variances in the emulsifiers’ composition are known. Variabilities in the formation of the foam and differing foam stability and firmness during storage and within the shelf life are identified problems [2–5]. Thus, robust and simple methods are required to control the composition and stability of E 471 emulsifiers in the dairy product.

According to Commission Regulation (EC) No 1333/2008 [6], E 471 emulsifiers are approved as food additives and are permitted to be used without a maximum limit; nevertheless, they should not be applied in higher amounts than reasonable. Regarding (EU) No 231/2012 [7], E 471 emulsifiers are mixtures of mono-, di- and triesters of fatty acids (FA) of edible oils with glycerol and additionally low amounts of free FA. According to this regulation, the quantity of the sum of mono- and diesters (= MAG and DAG) needs to be > 70% in the emulsifier product.

Emulsifiers of the type E 471 are industrially synthesized either by acylation of glycerol with FA or by transesterification of triacylglycerols (TAG) with glycerol [1]. The production process leads to product mixtures containing MAG and DAG in variable amounts and compositions. In addition, unprocessed educts (TAG, glycerol, and FA) are present in the mixtures. The production is difficult to control, why E 471 emulsifiers generally are mixtures with variable compositions and no standardized products are available.

In literature, methods for the extraction of MAG and DAG are reported, mainly focusing on the analysis of natural lipids (“lipidomics”), like blood and membrane lipids and animal and vegetable fats [8–13]. For sample preparation of lyophilized cells and human blood, treatment with sodium chloride solution and mixtures of chloroform/methanol were commonly applied [8, 10–13], while milk powder was simply dissolved in a mixture of *n*-hexane/*iso*-propanol [9]. Milk was extracted with a mixture of methylene chloride/methanol and the addition of sodium chloride [14]. All procedures should entirely extract MAG and DAG from lipoproteins; however, validation data showing the efficiency and reliability of the extraction methods, for example expressed as recoveries, was not presented. For analysis, mainly high-performance liquid chromatography coupled to mass spectrometry (HPLC–MS) [8–10, 12, 15] and gas chromatography coupled to MS (GC–MS) [16–19] were reported. For the analysis of MAG and DAG in vegetable oils and in E 471 emulsifiers, an AOCS standard GC method was published [20]. Thin-layer chromatography (TLC) methods for the separation and quantitation of neutral lipid classes like phospholipids and glycerides including MAG and DAG are also available [21–26]. Very recently, a screening method for the characterization of E 471 emulsifiers by high-performance thin-layer chromatography (HPTLC) was published [27]. Up to now, analysis of MAG and DAG of E 471 emulsifiers in food products has only been described for baked goods [28, 29] and margarines and mini-cakes [30] by HPLC–MS , but HPTLC-based methods generally were not reported. To the best of our knowledge, methods for the analysis of MAG and DAG of E 471 added to whipping cream and aerosol whipping cream were not yet described in literature.

The aim of the present study was to develop a suitable and simple screening method for the analysis of MAG and DAG from E 471 emulsifiers in aerosol whipping cream by HPTLC with fluorescence detection (FLD). Therefore, an effective and reliable procedure for the extraction of MAG and DAG from dairy matrix had to be developed. To compare different extractions, a model aerosol whipping cream containing known amounts of emulsifiers was analyzed. Separation of MAG and DAG from dairy matrix should easily be achieved by HPTLC without the need of a time-consuming clean-up step due to the great selection of solvents and separation techniques. For determination of MAG and DAG by FLD, the strategy according to Oellig et al. [27] was used, wherein the individual lipid classes are collectively detected and quantitated. For calibration, an emulsifier with known composition should be used. The developed method should be applied to commercially available aerosol whipping creams with labeled addition of E 471 to provide an overview of their composition.

## Material and methods

### Chemicals and materials

1-Stearoyl-*rac*-glycerol (> 99%), 1,2-distearoyl-*rac*-glycerol (> 99%), 1,3-distearoylglycerol (> 99%), stearic acid (> 99.5%, analytical standard grade), glyceryl tristearate (> 99%), 2-naphthoyl chloride (2-NCl) (98%), 4-(dimethylamino)pyridine (DMAP) (≥ 99%, reagent plus), primuline (dye content 50%), diethyl ether (≥ 99.5%, GC, puriss.), *n*-pentane (≥ 99% for residue analysis, Chromasolv), *tert*-butyl methyl ether (TBME, ≥ 99.8%, HPLC, Chromasolv), ethanol absolute (≥ 99.8%, HPLC, Chromasolv), methanol (LC–MS, Chromasolv), and methylene chloride (99.8%, anhydrous) were purchased from Sigma-Aldrich (Steinheim, Germany). Sodium hydrogen carbonate (NaHCO_3_, ≥ 99%, Ph. Eur., puriss.), tris(hydroxymethyl)aminomethane (TRIS, Pufferan ≥ 99.9%), chymotrypsin (≥ 1000 USP-U/mg, for biochemistry), and trypsin (5000 USP-U/mg) were obtained from Carl Roth GmbH & Co. KG (Karlsruhe, Germany). *n*-Hexane (95%, for pesticide residue analysis, Chemsolute) was purchased from Th. Geyer GmbH & Co. KG (Renningen, Germany). Formic acid (> 98%, analytical reagent grade) and hydrochloric acid (~ 37%) were obtained from Fisher Scientific (Schwerte, Germany). Ethane-1,2-diol (for synthesis) was from Merck (Darmstadt, Germany). Ultrapure water (> 18 MΩ cm) was supplied by a Synergy System (Merck Millipore, Darmstadt, Germany). HPTLC silica gel LiChrospher F_254_s plates from Merck were used without pre-washing.

Model aerosol whipping cream samples were produced by the Department of Soft Matter Science and Dairy Technology, University of Hohenheim (Stuttgart, Germany) according to the “Model aerosol whipping cream” section. Commercial whole milk, coffee cream, whipping cream, and aerosol whipping cream samples were bought in local supermarkets.

### Model aerosol whipping cream

Raw bovine milk provided by the research station Meiereihof (University of Hohenheim) was separated at a temperature of 60 °C using a separator (SA 10-T, Frautech SRL, Schio, Italy). Cream (> 30 g lipid/100 g sample) was heated to 90 °C with a batch pasteurizer (Pasteurisierer C600/45, Kälte Rudi, Keltern, Germany) and skim milk (≤ 0.1 g lipid/100 g sample) was pasteurized (72 °C for 28 s) by means of a plate heat exchanger (KS8FS1514, ATS-Südmo, Feldkirch, Germany). Cream was standardized to a lipid content of 30 g/100 g and heated to 80 °C in a metal beaker set in a water bath (Julabo Labortechnik, Seelbach, Germany). Emulsifier (400 mg/100 g) was added under constant stirring at a temperature ≥ 70 °C. After temperature equilibration (80 °C, 5 min), a pre-emulsion was prepared by dispersing the sample at 10,000 rpm for 3 min with a high-shear-blender (IKA, Staufen, Germany). Dispersing of the samples was conducted in a water bath (WarmMaster Deluxe, Merten&Storck, Drensteinfurt, Germany) set to 80 °C to prevent temperature loss during stirring. The sample was then homogenized with a two-stage homogenizer (APV-Gaulin, Lübeck, Germany) at a pressure setting of 6/0 MPa. The sample was collected in an 1-L laboratory bottle with high temperature screw caps (Schott, Mitterteich, Germany) and immediately cooled with ice water. Unhomogenized standardized model aerosol whipping cream (30 g lipid/100 g sample) without addition of emulsifier was used as reference sample. To ensure fat crystallization, the samples were stored at 5 °C for at least 24 h prior to HPTLC analysis.

### Standard solutions

#### Extraction method development

For quantitation of the native amount of DAG in whole milk, coffee cream, and whipping cream, a standard solution containing 1,2-distearin (1,2-DSt) in a concentration of 25 ng/μL in TBME was used. To determine the efficiency of investigated extraction procedures for MAG and DAG of E 471 from model aerosol whipping cream with E 471 (“Model aerosol whipping cream” section), standard solutions of the applied E 471 emulsifiers were prepared at concentrations of 20 ng/μL in TBME for application.

#### HPTLC method development

A combined standard stock solution was prepared by dissolving 4 mg of mono-, di-, tristearin, and stearic acid (MSt, DSt, TSt, and SA) in 10 mL of TBME. The stock solution was diluted 1:10 (*v/v*) with TBME for application, resulting in concentrations of 40 ng/μL for MSt, DSt, TSt, and SA.

Stock solutions of MAG and MAG/DAG emulsifiers were prepared in TBME at concentrations of 400 mg/L. The MAG emulsifier (97.8% MAG) comprised a mixture of MSt/monopalmitin (55:45) and the MAG/DAG emulsifier (59% MAG and 34.3% DAG) consisted of a mixture of C16 and C18 representatives with the following fatty acid composition: 43.3% of C16:0, 54.5% of C18:0, and 1.1% of C18:1 [5, 27]. For application, the emulsifier stock solutions were diluted with TBME to 40 ng/μL for the MAG and 80 ng/μL for the MAG/DAG emulsifiers.

#### Determination of limits of detection and quantitation

Determination of limits of detection and quantitation (LOD/LOQ) was done with a working standard-mix solution containing MSt and 1,2-DSt (1 ng/μL each) achieved by dilution from a combined standard stock solution containing MSt and 1,2-DSt (200 mg/L) with TBME.

#### Recovery experiments and sample analysis

To determine recovery of MAG and DAG for the final extraction procedure, the E 471 emulsifiers mentioned above were used. Emulsifier standards were individually prepared at a concentration of 16.7 mg/mL in a mixture of TBME/ethanol (1:1, *v/v*). The MAG emulsifier standard solution (16.7 mg/mL) was also used for calibration during the analysis of whipping cream and aerosol whipping cream samples from the German market.

### Internal standard preparation

For the preparation of the internal standard (ISTD) (1,2-bis-naphthoylethanediol), 0.8 g of 2-NCl and 2.4 g of DMAP were dissolved in 4.5 mL of methylene chloride in a 40-mL glass centrifuge tube equipped with a screw cap in an ultrasonic bath for 2 min. Five grams of ethane-1,2-diol were added, and the tube was briefly vortexed and stored for 1 week at 50 °C in a drying oven. After cooling to room temperature, 5 mL of *n*-hexane were added, and the tube was briefly vortexed. Excess of derivatization reagent was removed by twofold shaking with 7 mL of 2.5 M hydrochloric acid and twofold shaking with 7 mL of saturated NaHCO_3_ solution for 10 min on a small shaking device (VXR basic, IKA) at 2200 min^−1^. After each shaking step, centrifugation followed for 2 min at 3000 rpm and 18 °C (Heraeus Multifuge X1R, Thermo Scientific, Dreieich, Germany). The organic phase was transferred into a 12-mL screw-capped glass vial and the solvent was completely removed under a stream of nitrogen. The viscous residue was finally dissolved in 500 μL of TBME. The ISTD working solution was stored at room temperature.

### Extraction procedures

#### Simple liquid-liquid extraction

As samples, whole milk (lipid content of 3.8%), coffee cream (lipid content of 12%), and whipping cream (lipid content of 30%) were investigated. Liquid-liquid extraction (LLE) was performed in 6- or 12-mL glass centrifuge tubes equipped with screw caps. For the analysis of whole milk and coffee cream, 1 g of sample and for the analysis of whipping cream, 0.5 g of sample and 0.5 g of water were used. Either 1 mL of water or aqueous phosphoric acid (4%) was added, the tube was closed and vortexed, and LLE was done with different extraction solvents (*iso*-propanol, *iso*-propyl acetate, acetonitrile, ethyl acetate). The tube was shaken on a small shaking device at 2000 rpm^−1^ (VXR basic), the addition of sodium chloride followed, and the sample was again vigorously shaken. Different shaking times (10–30 min) were investigated and also the addition of *n*-hexane was tested. After centrifugation, aliquots of the diluted supernatant were used for high-performance thin-layer chromatography–fluorescence detection (HPTLC–FLD) according to Oellig et al. [27] to evaluate differences in matrix loads and extraction efficiency. Extraction efficiency for native DAG was evaluated by comparison of the signal response for the different procedures. For final comparison of LLE procedures, quantitation in whole milk, coffee cream, and whipping cream was done with a four-point calibration in the range of 50–500 ng 1,2-DSt/zone (“Extraction method development” section). For comparison, whole milk, coffee cream, and whipping cream were extracted according to the method of Röse-Gottlieb [31] and quantitation in the lipid fraction was done by HPTLC–FLD using the same calibration.

#### Enzymatic treatment

Model aerosol whipping cream with 400 mg E 471 emulsifier/100 g sample and without emulsifier (“Model aerosol whipping cream” section) were suspended in water and in TRIS buffer (50 mM, pH 8.2) in concentrations of 1 g sample/100 mL. One milliliter of the suspension was pipetted in a 6-mL glass centrifuge tube equipped with a screw cap and 100 μL of an aqueous solution of trypsin or chymotrypsin were added. Different ratios of protease to protein in the sample (1:7.5 and 1:15 (*w/w*)), digestion times (16–40 h, over one/two night/s), and temperatures (28–30 °C) were tested without and with slight shaking (300 min^−1^, KS 125, IKA). After enzymatic digestion, LLE was done with 2 mL of TBME for 20 min at 2200 min^−1^ on a small shaking device (VXR basic). After centrifugation, the clear supernatant (sample concentration 5 mg/mL) was transferred into a HPTLC vial and HPTLC–FLD was performed according to [27] to evaluate extraction efficiency. Recoveries from model aerosol whipping cream with E 471 were calculated by comparison of the signal response of MAG and DAG with those of the emulsifier standard in pure solvent with corresponding concentration (“Extraction method development” section), taking the native amount of DAG of model aerosol whipping cream (without E 471) into account.

### Final sample preparation

One gram of whipping cream was weighed into a 20-mL glass centrifuge tube equipped with a screw cap. After the addition of 40 μL of ISTD working solution, 3 mL of ethanol were added and the tube was gently shaken by hand for 5 s before it was further shaken for 30 min on a small shaking device (KS 125) at 250 min^−1^. Seven milliliters of water were added, the tube was briefly vortexed, and the addition of 2 mL of TBME followed. The tube was briefly vortexed again and stored for 20 min at room temperature. Finally, LLE was performed for 30 min on a small shaking device (VXR basic, IKA) at 2200 min^−1^. After centrifugation, an aliquot of the clear supernatant was diluted 1:100 (*v/v*) with TBME (sample concentration 5 mg/mL) and subjected to HPTLC analysis.

To determine the recovery of E 471 emulsifiers from aerosol whipping cream, model samples at a level of 400 mg emulsifier/100 g sample were investigated. As emulsifiers, an MAG and an MAG/DAG emulsifier (“Standard solutions” section) were applied. Model samples were processed according to the procedure described in “Model aerosol whipping cream” section (*n =* 5 for both emulsifiers on different days). Sample preparation was done as described above (*n =* 4). To verify native DAG in the samples, identically processed reference samples without emulsifier were used (*n =* 1). A four-point calibration of the applied MAG and MAG/DAG emulsifiers in the range of 200–600 mg emulsifier/100 g sample was used for quantitation. Therefore, 120–360 μL of the MAG and MAG/DAG emulsifier standards (“Recovery experiments and sample analysis” section) were pipetted into 20-mL glass centrifuge tubes, 40 μL of ISTD working solution were added, and the calibration standards were prepared according to the procedure described above for whipping cream. According to Oellig et al. [27], the lipid classes of MAG and 1,3-DAG were detected as the total and the amount was calculated with the respective calibration after peak areas have been normalized by concerning the ISTD.

### High-performance thin-layer chromatography–fluorescence detection

For HPTLC, primuline impregnated silica gel LiChrospher plates were used. Preparation was done according to the procedure recently described [27]. An Automatic TLC Sampler 4 (ATS4, CAMAG, Muttenz, Switzerland) was used for the application of sample and standard solutions as 6-mm bands on 20 cm × 10 cm plates and TBME was used as the rinsing solvent. For LOD/LOQ determination, the combined standard working solution containing MSt and 1,2-DSt (“Determination of limits of detection and quantitation” section) was applied in amounts of 1.5–20 ng/zone for MSt and 1,2-DSt, respectively. For recovery experiments and the analysis of whipping creams from the German market, the application volume generally was 10 μL. After application, the plate was dried for 10 min in a fume hood. Development was performed in the Automatic Developing Chamber (ADC2, CAMAG) with a mixture of *n*-pentane/*n*-hexane/diethyl ether (22.5:22.5:55, *v/v/v*) up to a migration distance of 70 mm. Before development, the plate activity was controlled by saturated magnesium chloride solution for 10 min (33% relative humidity). After the development, a drying period of 20 min followed inside a chamber in which the relative humidity was set to 47% by saturated potassium carbonate solution. Digital documentation under UV 254 nm and UV 366 nm illumination was done using the TLC Visualizer (CAMAG). For detection of the ISTD, the plate was scanned in absorption mode at UV 254 nm (deuterium lamp) by the TLC Scanner 4 (CAMAG) and for detection of MAG and DAG, the fluorescence mode was used at UV 366/> 400 nm (mercury lamp) with manual detector settings according to [27]. HPTLC instruments were controlled by the software winCATS, version 1.4.6.2002 (CAMAG).

### Sample analysis

Five aerosol whipping cream samples from the German market labeled with E 471 addition were analyzed (*n =* 4). The sample was conventionally taken from the pressurized container and the foam was stored in a glass beaker for 10 min before being weighed into the glass tube. Further sample preparation and HPTLC–FLD analysis were done according to “Final sample preparation” and “High-performance thin-layer chromatography–fluorescence detection” sections. The calibration range for the analysis of these purchased samples was extended to include low MAG and DAG contents. Thus, 30–360 μL of the MAG emulsifier solution (“Recovery experiments and sample analysis” section) were used for the sample preparation procedure according to “Extraction procedures” section, leading to 25–300 ng MAG per zone. Detection of the lipid classes MAG and DAG was done as described in [27]. For quantitation of MAG and DAG, the peak areas normalized to the ISTD were evaluated. Including the response factors of the C18:0 representatives of MAG and DAG, the quantities of the classes were calculated as C18:0 fatty acid and expressed as mg MAG and DAG per 100 g aerosol whipping cream, respectively.

## Results and discussion

A suitable method for the analysis of MAG and DAG of E 471 emulsifiers in aerosol whipping cream by HPTLC–FLD was developed. The chromatographic separation was optimized for whipping cream matrix and sample preparation methods for complete and reliable extraction of MAG and DAG from whipping cream were evaluated. Thereafter, validation of the entire method by LOD, LOQ, and recovery experiments took place. Finally, aerosol whipping cream samples from the German market were analyzed by HPTLC–FLD to determine and display the current application of E 471 emulsifiers.

### Sample preparation

The major intention of the present study was to develop a simple and reliable sample preparation method for a selective and quantitative extraction of MAG and DAG from dairy products. Co-extraction of interfering matrix components such as cholesterol should be avoided and the rearrangement of 1,2-/1,3-DAG should be omitted. In a first step, whole milk, coffee cream, and whipping cream were chosen to evaluate the extraction efficiency of native DAG. In further steps, emulsifier-free model aerosol whipping cream with a lipid content of 30% and a model aerosol whipping cream with 400 mg E 471 emulsifier/100 g sample were investigated. To extract MAG and DAG from dairy lipoproteins, LLE and enzymatic methods were tested, and the method according to Röse-Gottlieb [31] was used as a reference method. To verify the extraction success, initially, HPTLC–FLD according to [27] was used.

#### Liquid-liquid extraction

With the intention of a short extraction procedure, LLE was evaluated first. In literature, chloroform was often mentioned for the extraction of cells, human blood, and membrane lipids [8, 10–13, 32], and Fagan et al. used a solvent mixture containing methylene chloride for the extraction of lipids from milk [14]. To omit chlorinated toxic solvents, several alternative solvents were tested. Extractions with different extraction times, storing times, and the addition of salt and *n*-hexane for complete phase separation were verified (“Simple liquid-liquid extraction” section). Best efficiency for the extraction of native DAG from whole milk, coffee cream, and whipping cream was obtained by LLE with 3 mL of *iso*-propanol for 30 min after the addition of 1 mL of phosphoric acid (4%), a storing period of 5 min, and a further shaking for 10 min after the addition of sodium chloride and 1 mL of *n*-hexane. The extraction of native DAG from whole milk, coffee cream, and whipping cream showed reproducible results with RSD < 8% (*n =* 4 for each sample type). Quantitation of DAG in whole milk and coffee cream after fat extraction under alkaline conditions according to the reference method of Röse-Gottlieb [31] revealed results in the same order of magnitude with deviations between both methods < 10%. The results for the whipping cream, however, demonstrated that the simple procedure was not suitable for an entire extraction of DAG from the lipoproteins of this type of food. DAG amounts were distinctly lower (~ 20%) compared to the method of Röse-Gottlieb [31]. Hence, complete liberation of DAG from lipoproteins was not achieved by a simple LLE. Apart from this, the method according to Röse-Gottlieb [31] was not suitable for sample extraction because rearrangement of 1,2-/1,3-DAG occurred.

#### Enzymatic treatment

As an option for a higher extraction efficiency of DAG from the lipoproteins of whipping cream, enzymatic methods were investigated for model aerosol whipping cream with and without E 471 emulsifier. In literature, only a method for mini-cakes was reported [30]. Proteolytic digestion of the lipoprotein membrane should be achieved by using proteases and the entire lipid fraction should be released from the emulsion without rearrangement of 1,2-/1,3-DAG. Therefore, chymotrypsin and trypsin were used as proteases in different molar ratios and enzymatic digestion was evaluated after different reaction times and temperatures. Determination of MAG and DAG was performed after a simple LLE into TBME by HPTLC–FLD. Best recoveries of ~ 90% for both MAG and MAG/DAG emulsifiers were obtained with a molar ratio of trypsin/protein of 1:15 and a reaction time of 18 h. For DAG, however, rearrangement of 1,2-/1,3-DAG occurred, and recoveries for the sum of 1,2- and 1,3-DAG ranged between 180 and 210%, which could not be explained. Thus, enzymatic digestion with proteases turned out to be unsuitable for the reliable determination of DAG in whipping cream.

#### Optimization

Further attempts for the entire release of DAG from the lipoproteins of whipping cream were considered. Therefore, additional steps before LLE were evaluated. To correct volume errors during sample preparation, 1,2-bis-dinaphthoylethanediol (“Internal standard preparation” section) was used as ISTD.

##### Protein denaturation

For protein denaturation, the addition of urea, acetonitrile, and ethanol, also in combination with the addition of sodium chloride, was investigated. Various solvent to sample ratios and different procedures (storing, shaking, heating, and ultrasonication) and their duration were studied. Elevated temperature and ultrasonication did not enhance the efficiency of this step; however, the type of solvent and the solvent to sample ratio (tested ratios, 3:1 to 1:1) showed distinct effects. The addition of ethanol to the whipping cream turned out to release MAG and DAG from lipoproteins for both MAG and MAG/DAG emulsifiers best.

##### LLE into the organic phase

Next, LLE into TBME was optimized considering the addition of water, different solvent volumes, shaking times and intensities, and storing periods between the steps. During optimization, it became obvious that a storing period after the addition of water and prior to LLE distinctively enhanced the efficiency and the repeatability of the extraction.

Finally, slight shaking of whipping cream with ethanol, followed by the addition of water and LLE into TBME delivered recoveries close to 100% for MAG and DAG. MAG and DAG were detected in the fluorescent mode without matrix interferences.

##### Calibration standards

Emulsifier standards for calibration were treated the same way as the samples to guarantee identical conditions for both the standard solutions and the sample extracts. Because calibration in pure solvent and matrix-matched calibration (with emulsifier-free model aerosol whipping cream) showed identical calibration graphs (see Electronic Supplementary Material (ESM) Fig. S1), calibration in pure solvent was chosen for quantitation of MAG and DAG in whipping cream samples.

### High-performance thin-layer chromatography

**Fig. 1:**
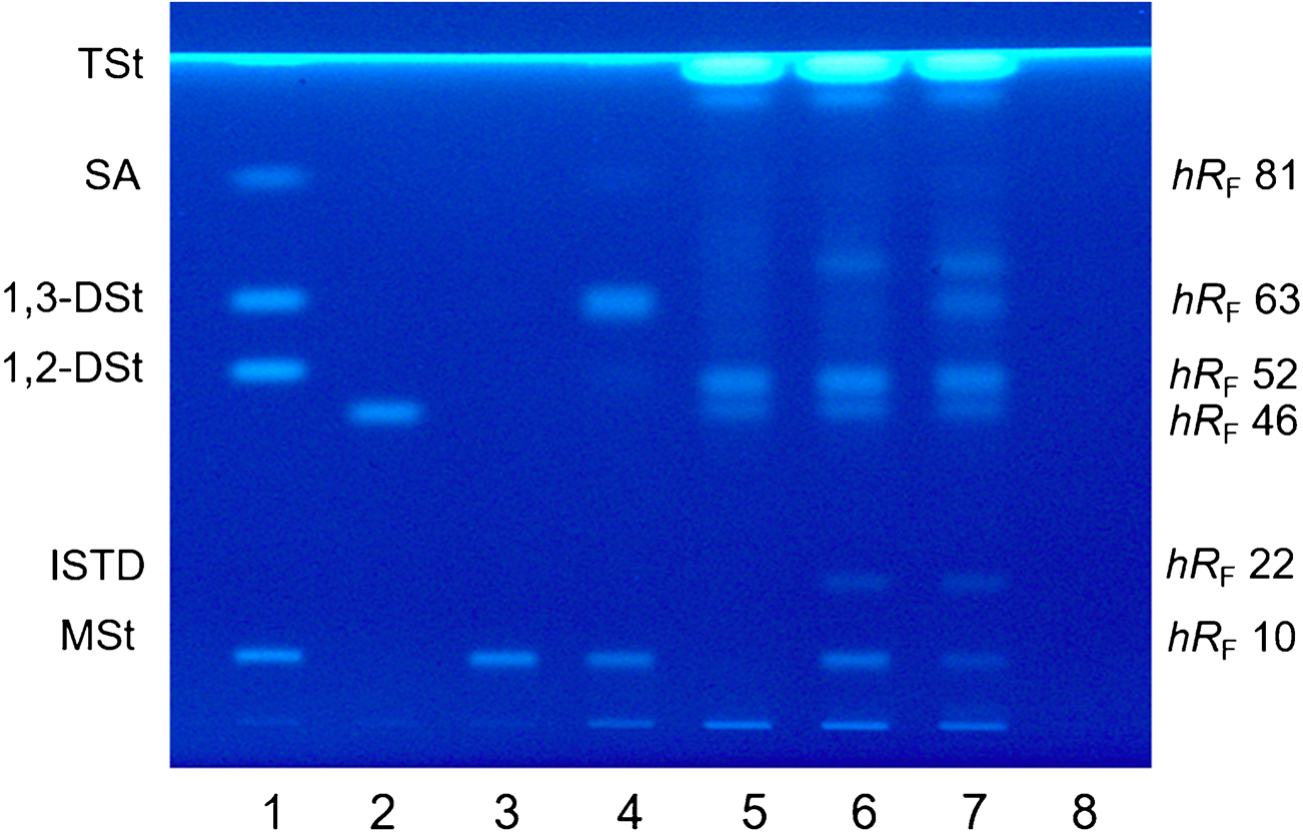
HPTLC chromatogram under UV 366 nm illumination after separation of (1) a standard-mix containing monostearin (MSt), 1,2-distearin (1,2-DSt), 1,3-distearin (1,3-DSt), stearic acid (SA), and tristearin (TSt) (each 400 ng/zone); (2) cholesterol (150 ng/zone); (3) an MAG emulsifier (400 ng/zone); (4) an MAG/DAG emulsifier (800 ng/zone); (5–8) from left to right, aerosol whipping cream samples without an emulsifier, with 400 mg MAG emulsifier/100 g including internal standard (ISTD), with 400 mg MAG/DAG emulsifier/100 g including ISTD, and a blank solvent sample. The ISTD was 1,2-bis-naphthoylethanediol. Chromatography was performed on primuline pre-impregnated LiChrospher silica gel plates by development with *n*-pentane/*n*-hexane/diethyl ether (22.5:22.5:55, *v/v/v*) to a migration distance of 70 mm. All samples were prepared according to the developed method, the application volume generally was 10 μL, and the sample amounts of aerosol whipping cream were 50 μg/zone

With the aim to separate MAG and DAG of E 471 from the dairy matrix, MAG, 1,2- and 1,3-DAG, FA, and TAG with fatty acid chains from C12:0–C18:1, an MAG and an MAG/DAG emulsifier, a cholesterol standard, and a model aerosol whipping cream extract were evaluated. Initially, the chromatographic system according to Oellig et al. [27], developed for the characterization of E 471 emulsifiers by fingerprints and to determine the lipid classes of the pure emulsifiers, was used and led to the successful separation of the dairy matrix from MAG, and thus, the interference-free detection of MAG. However, cholesterol, which is present in dairy lipids in remarkable quantities, co-migrated with 1,2- and 1,3-DAG and, therefore, resulted in their overestimation, hence hindered a reliable quantitation of DAG. To omit time-consuming sample clean-up procedures removing cholesterol from the matrix, which additionally can lead to isomerizations [14, 33, 34], the chromatographic separation of cholesterol from 1,2- and 1,3-DAG was investigated. Varying solvent ratios and further solvents (petroleum ether, *n-*heptane, TBME, diisopropyl ether) for the 2^nd^ development of the twofold development system according to Oellig et al. [27] were tested, but did not result in an entire separation of 1,2- and 1,3-DAG and from cholesterol, when mainly sharpness of the zones varied. Further method development was done with solvent mixtures well-known for the analysis of lipids by TLC [35–41] in slightly modified variations, i.e., without acidic components since the applied plate impregnation with primuline already contained formic acid. Moreover, irregular and spherical-shaped (LiChrospher) silica gel plates both pre-impregnated were investigated. Among the studied silica gel plates and solvent mixtures containing petroleum ether, *n*-pentane, *n*-hexane, *n-*heptane, diethyl ether, TBME, and diisopropyl ether in various combinations, LiChrospher plates and a mixture of *n*-pentane/*n*-hexane/diethyl ether were the most promising regarding sharpness of the zones and separation of analytes and matrix. After optimization of the solvent ratio and the developing distance, best separation of the lipid classes of MAG, 1,2-DAG, 1,3-DAG, and FA and from cholesterol was obtained with a single development applying a mixture of *n*-pentane/*n*-hexane/diethyl ether (22.5:22.5:55, *v/v/v*) up to a migration distance of 70 mm. Thereby, *hR*_F_ were 10, 52, 63, 81, and 46 for a mixture of MSt, 1,2-DSt, 1,3-DSt, SA, and a cholesterol standard (Fig. 1, 1–2). TAG migrated into the solvent front, which, however, was irrelevant because the quantitation of TAG was not necessary for the analysis of E 471 emulsifiers in whipping cream. Likewise, same *hR*_F_ were obtained for MAG and MAG/DAG emulsifiers (Fig. 1, 3–4). Interference-free detection and quantitation of MAG, 1,2-DAG, and 1,3-DAG in whipping cream was, therefore, guaranteed as exemplarily shown for an emulsifier-free model aerosol whipping cream and model aerosol whipping creams prepared with MAG and MAG/DAG emulsifiers (Fig. 1, 5–7). *hR*_F_ for the ISTD was 22 (Fig. 1, 6–7).

### Method validation

To verify method sensitivity, limits of detection and quantitation (LOD/LOQ) were determined for the C18:0 constituents MSt and 1,2-DSt because these MAG and DAG are the main components present in MAG and MAG/DAG emulsifiers. Determination of LOD and LOQ was performed according to the DIN 32645 [42] calibration method. For this method, at least five calibration standards close to the presumed LOD are used, showing a linear correlation between the amount of the analyte and the signal, when variance homogeneity between the LOD and the calibration solution with the highest concentration is required. Calculation of LOD and LOQ is based on the calibration equation and its quality and the applied calibration range. Calibrations were performed in the range 1.5–20 ng/zone of both MSt and 1,2-DSt, resulting in 3–40 mg MSt and 1,2-DSt per 100 g aerosol whipping cream (*n =* 4), taking the sample preparation into account (“Final sample preparation” section) and an application volume of 10 μL sample extract. Calibrations resulted in graphs of good linearity with high coefficients of correlation (*R*^2^ > 0.994). LOD and LOQ were determined to 1.8 and 5.7 ng for both MSt and 1,2-DSt/zone, corresponding to 4 and 11 mg MSt and 1,2-DSt per 100 g aerosol whipping cream. With RSD < 5%, the determination was well repeatable. Applying the response factors determined in previous work [27], which are for example equal for 1,2-DSt and 1,3-DSt, LOD and LOQ can be used for all representatives of the lipid classes of MAG and DAG. In any case, the developed HPTLC–FLD method allowed the quantitation of MAG and DAG amounts below the commonly applied quantity of ~ 400 mg E 471/100 g aerosol whipping cream.

Recovery experiments were performed with an MAG and an MAG/DAG emulsifier in model aerosol whipping cream containing 400 mg E 471/100 g sample (*n =* 4 for both emulsifiers and for each of the five replicates). Quantitation was done with the applied emulsifiers by means of a four-point calibration applying extracted calibration standards dissolved in pure solvent. The experiments were performed five times with different model aerosol whipping creams to additionally consider the variability of the composition of the model aerosol whipping cream and their differences in the production process. Model aerosol whipping cream with no addition of E 471 (reference sample) revealed MAG and 1,3-DAG contents below the LOQ, while native 1,2-DAG were present in high quantities (Fig. 1). The 1,2-DAG content, however, did not interfere the quantitation of MAG and 1,3-DAG. Besides, the applied MAG/DAG emulsifier only contained MAG and 1,3-DAG but no 1,2-DAG. Due to the high quantities of native 1,2-DAG, moreover, a quantitation of 1,2-DAG possibly originating from E 471 emulsifiers is not meaningful and was not further investigated. Regardless of this fact, the content of 1,2-DAG in whipping cream was generally determined (Emulsifiers in aerosol whipping creams from the German market).

Average recoveries for MAG and DAG from an MAG and an MAG/DAG emulsifier in model aerosol whipping cream ranged between 95 and 105% for MAG and 86 and 95% for DAG, respectively (Table 1). Intraday precision of recovery, expressed as RSD , with less than 7% (*n =* 4) for both lipid classes, showed the good repeatability of the entire sample preparation and the reliability of the method. Overall interday deviations below 5% for MAG from MAG and MAG/DAG emulsifiers and DAG from MAG/DAG emulsifiers (*n =* 5 days) confirmed the good repeatability of the extraction, independent of variations in the production of the model aerosol whipping cream. Thus, the suitability of the method was proven, also because no distinct loss of emulsifier during the extraction procedure was observed.

**Table 1:** Recoveries of MAG and DAG from model aerosol whipping cream at a level of 400 mg E 471 emulsifier/100 g, quantitated against the applied emulsifiers (solvent standards)

Production batch	Recovery in % ± SD^b^ (*n =* 4)				
	1	2	3	4	5
MAG emulsifier					
MAG	100.3 ± 3.6	103.5 ± 2.1	100.5 ± 2.7	95.3 ± 3.4	101.1 ± 1.1
MAG/DAG emulsifier					
MAG	102.3 ± 6.4	104.1 ± 3.4	104.6 ± 0.2	104.2 ± 2.0	105.4 ± 0.1
DAG^a^	92.4 ± 7.4	86.0 ± 3.2	94.6 ± 1.8	91.0 ± 1.4	90.7 ± 3.3

^a^DAG consisted of 100% of 1,3-DAG

^b^Standard deviation

### Emulsifiers in aerosol whipping creams from the German market

**Fig. 2:**
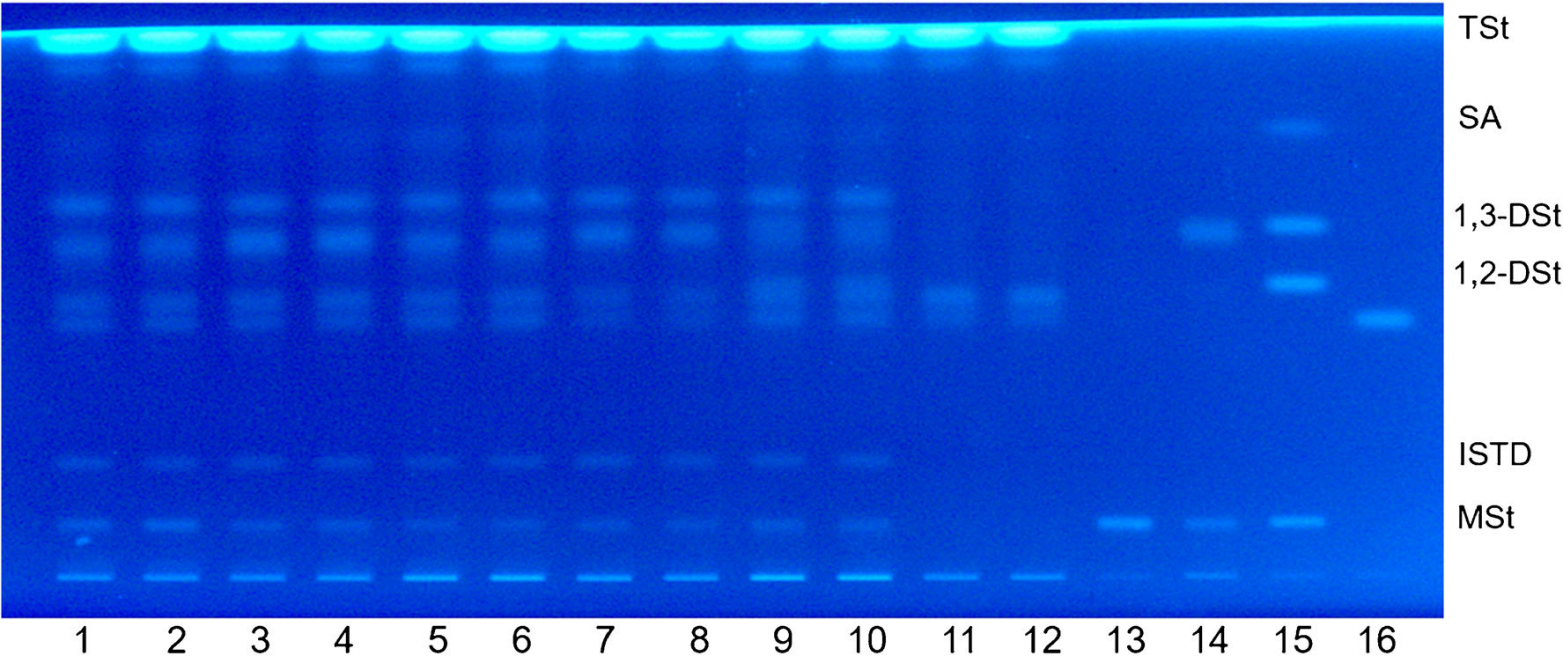
Separation/fingerprint of (1–10) five whipping cream samples with labeled E 471 addition from the German market prepared according to the developed sample preparation (*n =* 2, replicates were applied right next to each other); (11–12) two liquid whipping cream samples from the German market; (13–14) an MAG and an MAG/DAG emulsifier (200 ng/zone); (15) a standard-mix containing monostearin (MSt), 1,2-distearin (1,2-DSt), 1,3-distearin (1,3-DSt), stearic acid (SA), and tristearin (TSt) (200 ng/zone); and (16) cholesterol (100 ng/zone) on primuline pre-impregnated LiChrospher silica gel plates after development with *n*-pentane/*n*-hexane/diethyl ether (22.5:22.5:55, *v/v/v*) to a migration distance of 70 mm; plate image under UV 366 nm illumination. The internal standard (ISTD) was 1,2-bis-naphthoylethanediol. Sample amounts generally were 50 μg aerosol whipping cream/zone

Five aerosol whipping cream samples from the local market with labeled addition of E 471 were analyzed by the above described HPTLC–FLD method. Quantitation of the MAG and 1,3-DAG contents was performed with an MAG emulsifier (“Standard solutions” section) and results of both classes were calculated with the response factors for the respective C18:0 representatives according to Oellig et al. [27]. The visual fingerprint directly visualized both similarities and differences between the applied E 471 emulsifiers in the investigated samples (Fig. 2, 1–10). To identify the lipid class constituents, an MAG and an MAG/DAG emulsifier and a standard-mix of MSt, 1,2-DSt, 1,3-DSt, SA, and TSt were used (Fig. 2, 13–15). For comparison, the analysis of two liquid whipping cream samples (without E 471) showed the native constituents of dairy lipids like TAG, 1,2-DAG, and cholesterol; MAG and 1,3-DAG were not detected (Fig. 2, 11–12). All investigated aerosol whipping cream samples revealed the native constituents and additionally MAG and 1,3-DAG, when their ratio and absolute quantities varied considerably between the different samples (Fig. 2, 1–10). In samples 2 and 4, slightly higher *hR*_F_ for the 1,3-DAG zone were observed compared to samples 1 and 3, indicating a different fatty acid composition of the 1,3-DAG. Sample 5 showed a significant broader 1,3-DAG zone compared to the 1,3-DAG zone of the samples 1–4, which indicated a mixture of 1,3-DAG with different chain lengths. For all samples, the MAG contents ranged between 65 and 172 mg per 100 g aerosol whipping cream and the 1,3-DAG amounts between 279 and 501 mg per 100 g sample (Table 2). The E 471 quantities, calculated as the sum of MAG and 1,3-DAG, ranged from 384 mg/100 g in sample 5 to 610 mg/100 mg in sample 2. In general, the results for MAG and 1,3-DAG were well repeatable with RSD < 8% (*n* = 4). Summarizing, the detection of both MAG and 1,3-DAG in all samples showed that MAG/DAG emulsifiers are commonly used in aerosol whipping creams, and that MAG emulsifiers are rather seldom applied in this product.

**Table 2:** MAG and 1,3-DAG contents in five aerosol whipping creams from the German market

Sample	Mean content in mg/100 g whipping cream ± SD^a^ *(n =* 4*)*		
	MAG	1,3-DAG	Sum (MAG + 1,3-DAG)
1	172 ± 8	307 ± 16	479 ± 23
2	109 ± 8	501 ± 18	610 ± 26
3	65 ± 3	360 ± 22	426 ± 25
4	78 ± 6	436 ± 25	514 ± 30
5	105 ± 6	279 ± 23	384 ± 22

^a^Standard deviation

The analysis of the native content of 1,2-DAG in whipping cream samples from the German market with a lipid content of 30% (*n =* 12) revealed an average amount of 430 mg 1,2-DAG/100 g sample (*n =* 2 for each sample) with an overall RSD < 9%. The quantities matched well the amounts mentioned in literature [43], i.e., 440 mg 1,2-DAG/100 g sample (lipid content of 30%).

## Conclusions

HPTLC–FLD was shown as a reliable and efficient method for the analysis of MAG and DAG of E 471 emulsifiers in aerosol whipping cream. Treatment with ethanol and LLE into TBME as sample preparation were directly followed by HPTLC. Time-consuming clean-up procedures for the separation of interfering constituents like TAG and cholesterol were redundant. Determination by FLD on primuline impregnated plates was performed with an emulsifier with known content of MAG as calibration standard and the individual lipid classes were collectively detected and quantitated. The sensitivity with LOD and LOQ for MAG and DAG of 4 and 11 mg/100 g aerosol whipping cream, respectively, guaranteed the reliable determination of E 471 emulsifiers below the commonly applied quantity of ~ 400 mg emulsifier per 100 g sample. Recoveries close to 100% with low relative standard deviations were obtained for MAG and DAG from model aerosol whipping cream with an addition of 400 mg E 471 emulsifier per 100 g sample. In aerosol whipping creams from the German market with labeled E 471 addition, exclusively MAG/DAG emulsifiers were present, and quantities ranged between 384 and 610 mg/100 g sample.

## Electronic supplementary material

ESM 1 (PDF 160 kb)
