# Supplementary material for: Determination of mono- and diacylglycerols from E 471 food emulsifiers in aerosol whipping cream by high-performance thin-layer chromatography–fluorescence detection
Source: Anal Bioanal Chem. 2020 Aug 30;412(27):7441–51. doi: 10.1007/s00216-020-02876-2 (PMC7533252; doi:10.1007/s00216-020-02876-2)
Supplement: Supplementary file 1 — (PDF 160 kb) [file 216_2020_2876_MOESM1_ESM.pdf]

Analytical and Bioanalytical Chemistry

Electronic Supplementary Material

Determination of mono- and diacylglycerols from E 471 food emulsifiers in aerosol whipping cream by high-performance thin-layer chromatography–fluorescence detection

Claudia Oellig, Max Blankart, Jörg Hinrichs, Wolfgang Schwack, Michael Granvogl

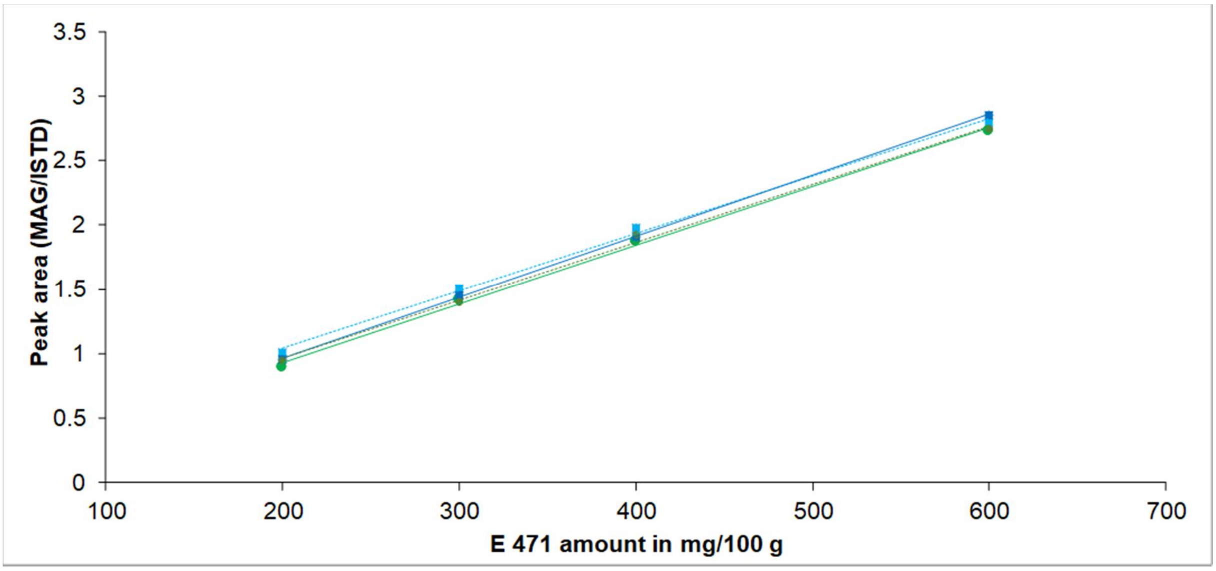

Fig. S1 Four-point calibration graphs (200–600 mg MAG/100 g sample) in pure solvent (*n* = *2*) compared to matrix-matched calibration graphs (*n* = *2*) of MAG from an MAG emulsifier. All calibration standards were prepared according to the developed method and the application volume was 10 µL. Light and dark blue squares and continuous and dashed lines show calibration graphs in pure solvent. Light and dark green circles and continuous and dashed lines correspond to matrix-matched calibration graphs
